# Progress and Character of Cholera

**Published:** 1854-06

**Authors:** 


*Progress and Character of Cholera.*

IN another part of this number of the Journal, will be found some account of the cholera, so far as it has prevailed in this city up to June 13th, 1854. We have only to add that since that time and up to the present date, June 28th, the prevalence of the disease has not increased.

As near as we can ascertain both from observation and inquiry, the disease first made its appearance in the city during the last week in April. Isolated cases continued to occur from that time to the first of June. From the 9th inst to the present time an average of 4 or 5 deaths per day have occurred from this disease. One half of these have been *immigrants* just arrived from the Atlantic; and nearly all of the other half have been from that class of foreign born laborers who live in the worst possible style and make free use of beer, whisky and brandy. We have heard of only two or three deaths amongst our American citizens, from cholera, during the present year. Keeping in view the fact that we have a population of about 70,000, it will be seen that the number of cases have been comparatively very few, and it is evident that the disease has not assumed anything like an *epidemic form;* although many of those which have occurred have been as severe and rapidly fatal as in any former year.

During the last week, a less number of cases of well marked cholera have come under our own observation, and a larger num-

Page(s) missing

